# The impact of manganese on vascular endothelium

**DOI:** 10.1007/s43188-024-00260-1

**Published:** 2024-08-13

**Authors:** Gustavo H. Oliveira-Paula, Airton C. Martins, Beatriz Ferrer, Alexey A. Tinkov, Anatoly V. Skalny, Michael Aschner

**Affiliations:** 1 Department of Molecular Pharmacology, Albert Einstein College of Medicine, Bronx, NY 10461 USA; 2 Laboratory of Ecobiomonitoring and Quality Control, Yaroslavl State University, Yaroslavl, 150003 Russia; 3 IM Sechenov First Moscow State Medical University (Sechenov University), Moscow, 119435 Russia

**Keywords:** Manganese, Endothelial cells, Blood–brain barrier, Neurotoxicity, Oxidative stress

## Abstract

Manganese (Mn) is an essential trace element involved in various physiological processes, but excessive exposure may lead to toxicity. The vascular endothelium, a monolayer of endothelial cells within blood vessels, is a primary target of Mn toxicity. This review provides a comprehensive overview of the impact of Mn on vascular endothelium, focusing on both peripheral and brain endothelial cells. In vitro studies have demonstrated that high concentrations of Mn can induce endothelial cell cytotoxicity, increase permeability, and disrupt cell–cell junctions through mechanisms involving oxidative stress, mitochondrial damage, and activation of signaling pathways, such as Smad2/3-Snail. Conversely, low concentrations of Mn may protect endothelial cells from the deleterious effects of high glucose and advanced glycation end-products. In the central nervous system, Mn can cross the blood–brain barrier (BBB) and accumulate in the brain parenchyma, leading to neurotoxicity. Several transport mechanisms, including ZIP8, ZIP14, and SPCA1, have been identified for Mn uptake by brain endothelial cells. Mn exposure can impair BBB integrity by disrupting tight junctions and increasing permeability. In vivo studies have corroborated these findings, highlighting the importance of endothelial barriers in mediating Mn toxicity in the brain and kidneys. Maintaining optimal Mn homeostasis is crucial for preserving endothelial function, and further research is needed to develop targeted therapeutic strategies to prevent or mitigate the adverse effects of Mn overexposure.

**Graphical Abstract:**

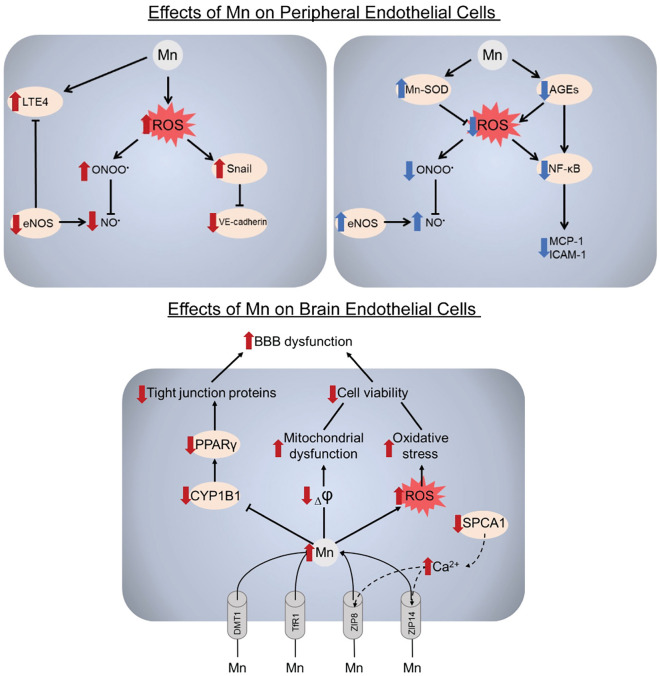

## Introduction

Numerous trace elements play indispensable roles in sustaining physiological processes, with approximately 30 elements deemed essential for life and vital for regulating biological functions. These essential elements are intricately involved in cellular metabolism, enzyme activities, and overall health maintenance. Among them are essential metals like iron, zinc, potassium, sodium, and manganese (Mn), each contributing uniquely to various physiological pathways [1]. For instance, iron is indispensable for oxygen transport in the blood [2], zinc plays a pivotal role in enzymatic reactions and immune function [3], while potassium and sodium are essential for nerve signaling and muscle contraction [4]. Mn, on the other hand, is essential for antioxidant defense mechanisms and bone development as well as it is involved in a variety of physiological processes [5]. Nevertheless, excessive exposure to Mn can result in toxic effects, manifesting in various health complications. For instance, it has been linked to the development of neurodegenerative disorders, such as Alzheimer’s and Parkinson’s diseases [6]. Additionally, prolonged exposure to Mn has emerged as a potential risk factor for hepatic illnesses, compromising liver function and overall metabolic health [7]. Moreover, studies have indicated its association with cardiovascular diseases, including hypertension and atherosclerosis [8, 9].

The endothelium, a monolayer of endothelial cells within blood vessels, plays a pivotal role in ensuring the maintenance of normal vascular functions. This multifaceted tissue, not only governs the permeability between blood and tissues, but also orchestrates the tone of vascular smooth muscles [10]. It achieves this intricate balance by facilitating the release of various vasoactive substances, including nitric oxide (NO), which acts as a potent vasodilator, relaxing blood vessels and promoting optimal blood flow throughout the body. Additionally, the endothelium exerts control over other vasoactive agents, such as endothelin and prostacyclin, further influencing vascular tone and hemodynamic stability [11]. In the central nervous system, the endothelium plays a vital role in forming and maintaining the blood–brain barrier (BBB), a highly selective barrier that regulates the exchange of substances between the brain and bloodstream [12]. BBB dysfunction has been implicated in various neurological disorders, including Alzheimer’s and Parkinson’s diseases, cerebral ischemia, and epilepsy [13, 14]. As Mn can cross the BBB and promote neurotoxicity [15], understanding the mechanisms of Mn uptake by brain endothelial cells and its impact on BBB integrity is crucial for elucidating the pathogenesis of Mn-induced neurotoxicity.

This review aims to provide a comprehensive overview of the impact of Mn on vascular endothelium, focusing on both peripheral and brain endothelial cells. We discuss the mechanisms of Mn uptake by endothelial cells, the effects of Mn exposure on endothelial function and integrity, and the role of endothelial dysfunction in mediating Mn toxicity in various organ systems. Furthermore, we highlight the importance of in vivo studies in validating the findings from in vitro models. Throughout the review, unless otherwise stated, the abbreviation ‘Mn’ refers to Mn2+, as it is the most common and stable oxidation state of Mn in biological systems [16].

## Manganese: general aspects

Manganese ranks as the 12th most abundant element in the Earth’s crust, distributed through natural processes like water and wind erosion, as well as human activities such as mining and industrial production [17]. This trace element plays a vital role in numerous enzymatic processes, energy metabolism, cell function maintenance, blood sugar regulation, reproduction and development [18, 19]. Typically, healthy levels of Mn range from 6 to 12 μg/L in whole blood, 0.4 to 0.85 μg/L in serum, and 1 to 8 μg/L in urine [20].

Maintaining sufficient Mn levels is easily achievable through dietary sources rich in the mineral, such as seeds and vegetables, making manganese deficiency rare compared to overexposure [5]. However, excessive Mn intake can lead to toxicity, typically occurring when levels exceed about three times the physiological concentration [21]. Toxic levels can arise from overexposure through ingestion or inhalation of high manganese concentrations, or in cases of iron deficiency, where shared transport mechanisms between iron and Mn lead to its bioaccumulation [22, 23]. Additionally, impaired Mn excretion due to hepatic dysfunction can contribute to toxic buildup within the body [7].

Individuals may be exposed to Mn by two different routes of exposure: non-occupational (environmental exposure) or occupational exposure. The primary non-occupational route of Mn exposure comes from consuming food rich in Mn or ingesting contaminated water, especially in geological regions where rocks containing high levels of Mn seep into aquifers, leading to elevated Mn concentrations in groundwater [5, 24]. Moreover, environmental contamination caused by the advancement of the Mn industry has sparked concerns about environmental pollution, which could lead to excessive Mn exposure and raises concerns about potential Mn overexposure for the general public. Particularly, those living near mining, manufacturing, and welding facilities face the highest risk of Mn toxicity, even without direct occupational exposure [25]. Increased atmospheric Mn levels in areas surrounding factories engaged in Mn production have been noted. On the other hand, Mn exposure can also occur in various industrial operations, including mining, welding, ferroalloy production, smelting, and battery manufacturing [26, 27]. Particularly, Mn-containing fumes and dust in industrial environments pose a significant risk of enhanced exposure. Chronic inhalation of high levels of airborne particulates may lead to elevated Mn levels in the body [27, 28].

Mn toxicity impacts numerous vital biological pathways, spanning from oxidative stress and endoplasmic reticulum stress to apoptosis, inflammation, cell signaling, and neurotransmitter metabolism. Emerging studies highlight Mn’s influence on the transcriptional regulation of these pathways upon exposure. Autophagy serves as a critical defense mechanism against Mn-induced toxicity; however, Mn can also instigate autophagic flux and dysfunction, especially under conditions of low Mn levels [29]. This intricate interaction between Mn and autophagy may interconnect with mitochondrial dysfunction, endoplasmic reticulum stress, and apoptosis, presenting a multifaceted picture of Mn toxicity [30–32]. Moreover, a recent study demonstrate that the acetylation of β-catenin induced by Mn impeded its movement into the nucleus, leading to a decrease in Pax6 expression, thereby disrupting the differentiation of neural stem cells [33].

Mn impact on the brain and its association with neurodegenerative diseases such as Alzheimer’s and Parkinson’s is well-recognized [6]. Additionally, it affects vital organs such as the liver and heart. A recent study highlighted significant differences in Mn levels in long-term cholestasis patients compared to the control group, suggesting cholestatic pathologies may contribute to Mn accumulation and subsequent toxicity [34]. Similarly, research by Long et al. revealed elevated Mn levels in both whole blood and the globus pallidus of patients with liver cirrhosis and chronic hepatic encephalopathy [35]. Animal studies indicate Mn’s rapid accumulation in heart tissue, leading to acute or sub-acute cardiovascular disorders such as cardiodepression and hypotension. These toxic effects are likely linked to Mn-induced mitochondrial damage and its interaction with calcium channels in the cardiovascular system [36]. Therefore, the widespread presence of Mn in the environment and its ability to accumulate in and adversely impact multiple organ systems, including the brain, liver, and cardiovascular system, highlights the critical need to better understand the mechanisms underlying Mn toxicity and develop effective strategies to mitigate its detrimental effects on human health.

## An overview of the vascular endothelium

### Importance of the endothelium for cardiovascular homeostasis

The vascular endothelium, situated as the innermost layer of blood and lymphatic vessels, covers the entire surface of the vascular system, serving as the crucial interface between circulating blood or lymph and the vessel wall [37, 38]. Initially perceived as a passive layer that merely facilitated fluid circulation, it was discovered in the nineteenth century to be a single cell thick [39]. Florey, in his early investigations into the ultrastructure of the vascular endothelial cell, predicted the possibility of groundbreaking discoveries through the study of these cells, challenging the prevailing notion that the endothelium was merely analogous to cellophane wrapping [40]. Indeed, over the past decades, research into the vascular endothelium has yielded significant productivity, greatly enhancing our comprehension of the normal functioning of the vasculature [41]. These studies revealed that, in addition to serving as a barrier between the blood and the surrounding tissue of the body, the endothelium is a dynamic structure that plays critical roles in cardiovascular homeostasis by producing various factors crucial for cell adhesion, thrombosis, thrombolysis, platelet and leukocyte interaction with the vascular wall, smooth muscle cell proliferation, and the regulation of vascular tone [41].

A functioning endothelium serves as a barrier, limiting the passage of diverse molecules from cells and blood into the sub-endothelial space [37, 38]. To meet metabolic demands, specific mechanisms facilitate the transport of essential circulating macromolecules across endothelial cells [41]. Tight junctions and transporters play pivotal roles as selective barriers for circulating macromolecules such as glucose and amino acids [42]. The endothelium also actively participates in host defense and inflammation by releasing chemokines, which influence various immune cells such as monocytes, neutrophils, eosinophils, and lymphocytes [41]. The role of endothelial cells in host defense and inflammation also involves the regulation of the movement of leukocytes into tissues through a meticulously controlled process involving adhesion molecules [38, 43]. These molecules facilitate the binding of leukocytes to the endothelium by interacting with specific ligands on the leukocytes [38, 43]. Endothelial cells express various adhesion molecules including E-selectin, P-selectin, intercellular adhesion molecule, and vascular cell adhesion molecule [44]. The expression of these molecules allows the movement of leukocytes through endothelial cells, maintaining endothelial integrity, while allowing activated inflammatory cells to migrate to sites of infection or injury [44].

The endothelium also plays a critical role in both thrombosis, the formation of blood clots, and thrombolysis, the breakdown of blood clots [45]. As a barrier, endothelium prevents blood clotting factors from coming into contact with subendothelial prothrombotic extracellular matrix components [45]. In addition, the endothelium maintains a delicate balance between pro-thrombotic and anti-thrombotic factors to prevent inappropriate clot formation while ensuring the integrity of the vascular system [46]. Dysfunction of the endothelium can disrupt this equilibrium, leading to cardiovascular complications such as venous thromboembolism, arterial thrombosis, and myocardial infarction [47]. In thrombosis, endothelial dysfunction or damage can lead to the exposure of subendothelial collagen and tissue factor, triggering platelet activation and adhesion [45]. Endothelial cells also release von Willebrand factor, which promotes platelet adhesion to the exposed collagen through the glycoprotein Ib-IX-V receptor complex [46]. On the other hand, the endothelium also produces several anticoagulant and fibrinolytic factors that prevent excessive clot formation. Endothelial cells express thrombomodulin, which binds thrombin and enhances the activation of protein C, a potent anticoagulant that inhibits coagulation factors Va and VIIIa [44]. Moreover, endothelial cells secrete tissue plasminogen activator, which converts plasminogen into plasmin, the enzyme responsible for degrading fibrin strands within the clot, leading to its dissolution [46]. This is essential for restoring blood flow and preventing complications such as tissue ischemia and organ damage, ultimately protecting the cardiovascular system [48].

In addition to the mechanisms described above, the endothelium also plays a significant role in cardiovascular homeostasis by producing various vasoactive mediators that regulate vascular tone [41]. NO, prostacyclin, and endothelin are potent vasoactive substances released from the endothelium in response to both humoral and mechanical stimuli [44]. These substances have a profound impact on both the function and structure of the underlying vascular smooth muscle [44]. NO is a small, gaseous, and lipophilic molecule produced from L-arginine by three distinct synthases: neuronal (NOS1), endothelial (NOS3), and inducible (NOS2) NO synthases [11, 49]. Under normal physiological conditions, NOS3 primarily generates endothelium-derived NO, making it a key player in cardiovascular homeostasis [50]. This significance has been consistently shown in both clinical and experimental investigations [50]. For instance, studies using NOS3 knockout mice reveal that these mice are hypertensive and have increased susceptibility to stroke and other cardiovascular alterations [51]. Similarly, inhibiting NOS3 pharmacologically yields comparable alterations to those observed in NOS3-deficient mice, including elevated blood pressure [52].

Once produced by NOS3 in the endothelium, NO diffuses through platelet or vascular smooth muscle cell membranes, where it activates the soluble guanylate cyclase, an enzyme that converts guanosine triphosphate into cyclic guanosine monophosphate (cGMP) [53–55]. While this process reduces platelet reactivity [56], elevated cGMP levels activate protein kinase G-1 in vascular smooth muscle cells, triggering the phosphorylation of various cellular proteins and reducing intracellular free calcium levels, thereby promoting vascular relaxation [57]. As intracellular calcium influx is known to stimulate the proliferation of vascular smooth muscle cells, NO exerts antiproliferative effects via cGMP-dependent inhibition of calcium influx, which further contributes to maintaining cardiovascular homeostasis [58].

NO can also indirectly influence vascular tone and proliferation by regulating the redox environment of vascular cells [56]. This effect involves the antioxidant properties of NO, which are attributed to its scavenging of superoxide anion, as well as the upregulation of the antioxidant enzyme superoxide dismutase [59]. Superoxide dismutase catalyzes the dismutation of superoxide anion to hydrogen peroxide [59]. However, NO reaction with superoxide anion can lead to peroxynitrite production [60], which is typically limited by relatively high concentrations of superoxide dismutase [61]. However, when NO levels increase, such as due to NOS2 upregulation, the formation of peroxynitrite may prevail, leading to impaired vascular homeostasis and endothelial dysfunction, which is a major feature in cardiovascular diseases [62].

Like NO, prostacyclin is another endothelium-derived vasoactive substance that serves as a potent vasodilator and inhibitor of platelet aggregation [63]. The synthesis of prostacyclin begins with the liberation of arachidonic acid from membrane-bound lipids by phospholipase A2, a process dependent on calcium in endothelial cells [64]. Arachidonic acid is ultimately metabolized to prostaglandin H2, which serves as the substrate for prostacyclin synthetase, leading to prostacyclin production [64]. Once produced, prostacyclin primarily acts on prostacyclin receptors (IP), which are G-protein-coupled receptors found in tissues throughout the body, particularly enriched on platelets and vascular smooth muscle cells [63, 64]. Activation of IP receptors leads to G-protein-mediated activation of adenylate cyclase, which in turn catalyzes the formation of cyclic adenosine monophosphate (cAMP) [65]. Elevated cAMP levels subsequently phosphorylate protein kinase A, ultimately resulting in reduced calcium levels in most target cells, including vascular smooth muscle cells and platelets [64]. This induces relaxation of vascular smooth muscle cells, resulting in vasodilation and consequent reduction in peripheral vascular resistance, thereby lowering blood pressure [66]. Reduced calcium levels in platelets induces the suppression of platelet activation pathways and decreased platelet aggregation [67]. By mitigating platelet aggregation, prostacyclin effectively prevents the formation of blood clots within the vasculature, thereby reducing the risk of thrombotic events associated with cardiovascular complications [67].

Endothelin is another vasoactive substance produced by endothelial cells, exerting marked effects on vascular tone and contributing significantly to cardiovascular homeostasis [44]. While there are three types of endothelin (ET-1, ET-2, and ET-3), vascular endothelial cells primarily produce ET-1 [68], which exerts vasoconstrictor actions by stimulating ETA receptors located on vascular smooth muscle cells [44]. This stimulation leads to vasoconstriction, resulting in increased vascular resistance and blood pressure [44]. Conversely, endothelin also has vasodilator actions mediated through the stimulation of ETB receptors present on endothelial cells [69]. Activation of ETB receptors induces the release of vasodilatory substances such as NO and prostacyclin, promoting vasodilation and counteracting the vasoconstrictor effects of ET-1 [70]. The intricate balance between endothelin-induced vasoconstriction and vasodilation plays a crucial role in regulating vascular tone and maintaining cardiovascular homeostasis [71]. Dysregulation of endothelin signaling has been implicated in various cardiovascular disorders, including hypertension, atherosclerosis, and heart failure [71].

### Importance of the endothelium for the central nervous system

While the brain is physically separate from the general blood circulation, it relies on tightly regulated communication with circulating blood to maintain its dynamic activity. This communication is facilitated by a specialized network of brain vasculature [72, 73]. Similar to peripheral blood vessels, endothelial cells in the brain interact with pericytes and smooth muscle cells to form the basic structure. However, unlike other tissues, the brain vasculature goes a step further by incorporating astrocytes, microglia, and neurons. This unique association forms the neurovascular unit, a critical structure responsible for maintaining both cerebral homeostasis and the blood–brain barrier [74, 75]. Under physiological conditions, the endothelium regulates the delivery of oxygen and nutrients to the brain, as well as vascular permeability, blood flow, and immune cell trafficking. This coordinated regulation helps maintain brain homeostasis [76, 77].

The endothelium makes critical contributions to maintain brain homeostasis, including the BBB formation and integrity. The BBB is a highly selective barrier that meticulously controls the exchange of substances between the brain and bloodstream. This vital function protects the central nervous system from pathogens, toxins, and other harmful substances while allowing essential nutrients like oxygen and glucose to pass through [78, 79]. The BBB is a highly specialized structure composed of several key elements: endothelial cells, astrocytic end-feet, and pericytes [73, 80]. Endothelial cells tightly regulate the passage of substances between blood and brain tissue. Astrocyte end-feet, in turn, surround the endothelium, providing structural support and contributing to BBB function through signaling molecules. Finally, pericytes wrap around the blood vessels, offering additional stability and contributing to the regulation of both blood flow and BBB function.

The endothelial cells from BBB are connected by tight junctions and adherens junctions, which play a critical role in BBB permeability. These specialized protein complexes bind endothelial cells together, severely restricting the passage of most substances between the bloodstream and the brain protecting the delicate neural environment. Occludin holds the distinction of being the first protein identified exclusively within tight junctions [81]. While the full range of its functions remains under investigation, studies using occludin-deficient mice offer some clues. These mice exhibited altered calcium concentrations within the brain despite normal serum calcium levels [82]. This finding suggests that occludin may play a crucial role in maintaining the integrity of BBB tight junctions. Tricellulin, another protein residing within tight junctions, appears to step in and partially compensate when occludin function is compromised. This ability likely stems from their structural similarity [83, 84]. Tricellulin contributes to the overall impermeability of the BBB by increasing paracellular electrical resistance and blocking the passage of macromolecules Interestingly, tricellulin achieves this feat without affecting the permeability of ions [85, 86]. This selectivity ensures essential nutrients can still reach the brain while keeping out potentially harmful substances.

Another crucial component of BBB tight junctions is a family of proteins called claudins. Among these, claudin-5 holds particular importance. It’s specifically enriched in the brain’s endothelial cells [87]. Claudin-5 deficient mice die within 10 days after birth. Interestingly, their blood vessels develop normally, and they do not exhibit any signs of bleeding. However, a critical flaw is revealed in the BBB—it becomes more permeable, especially for small molecules less than 400 kDa in size [88]. This increased permeability suggests that claudin-5 plays a vital role in forming a functional BBB. Junctional adhesion molecules (JAMs) are a class of type-I transmembrane glycoproteins belonging to the immunoglobulin superfamily [89]. Within the BBB, these proteins play a crucial role in formation and maintenance of tight junctions and regulation of paracellular permeability [89–91].

Whereas tight junctions restrict the paracellular movement of most solutes, including ions and small molecules. In contrast, adherens junctions, primarily mediated by vascular endothelial-cadherin (VE-cadherin), promote endothelial cell interaction and maintain the integrity of the blood–brain barrier [92]**.** VE-cadherin is a transmembrane protein crucial for adherens junction function and plays a critical role in maintaining BBB integrity [93, 94]. VE-cadherin deficiency in mice disrupts the localization of key tight junction proteins [95]. These findings suggest that VE-cadherin is essential for maintaining a functional BBB through its role in adherens junctions.

Emerging evidence suggests that BBB dysfunction is a contributing factor in various neurological disorders, including Alzheimer’s disease [14, 96, 97], Parkinson’s disease, cerebral ischemia, and epilepsy [13]. This disruption compromises the BBB’s role in restricting the passage of harmful substances, such as microbial pathogens and toxic molecules, into the brain [79, 80]. Consequently, BBB dysfunction can trigger neuroinflammatory responses, potentially accelerating neurodegeneration. Therefore, therapeutic strategies aimed at maintaining BBB integrity hold promise in delaying or preventing the progression of neurodegenerative diseases.

The endothelium also plays a critical role in regulating the passage of immune cells and molecules into the central nervous system. Under normal conditions, the BBB tightly controls substance entry to protect the neuronal environment. However, during neuroinflammation, activated endothelial cells can increase BBB permeability, allowing immune cells and inflammatory mediators into the brain [98, 99]. Neuroinflammation, a response to pathogen infiltration or neuronal damage, involves the interplay of immune response and the nervous system, where endothelial cells play a crucial role. This response activates resident immune cells within the CNS, such as astrocytes and microglia [100, 101], as well as recruits peripheral immune cells like T cells and macrophages [102, 103].

Endothelial cells contribute to neuroinflammation through several distinct mechanisms. In healthy states, the endothelium acts as a barrier, hindering uncontrolled immune cell access into the brain [104]. Under neuroinflammatory conditions, activated endothelial cells become leaky, increasing BBB permeability, and allowing the entrance of immune cells and inflammatory molecule entry into the brain tissue [105]. Additionally, activated endothelial cells produce cytokines, chemokines, and adhesion molecules, amplifying the immune response [106–109]. Endothelial cells also aid leukocyte recruitment in infection, injury, or autoimmunity by upregulation endothelial adhesion molecules at the junctions of the BBB. Thus, T cells are allowed to pass the BBB by increased expression of adhesion molecules like ICAM-1 and VCAM-1 on the endothelial cells [110, 111].

## Effects of Mn on endothelial cells

### Effects of Mn on peripheral endothelial cells

Endothelial cells are essential for maintaining the integrity of the renal glomerular filtration barrier, preventing the leakage of proteins and blood cells into the urine, and ensuring proper renal function [112]. Approximately 5% of circulating Mn is distributed to the kidneys [113], where it undergoes filtration by the glomerular filtration barrier, composed of glomerular endothelial cells [114]. Dysfunction of these endothelial cells compromises the glomerular filtration barrier, resulting in conditions such as proteinuria and hematuria, which are indicative of renal failure [112]. Since cases of acute renal failure have been reported following accidental ingestion of Mn [115], Gao et al. investigated whether this metal perturbs renal glomerular endothelial cells [116]. Interestingly, the authors found that Mn exposure (0.2 mM) increased the permeability of human renal glomerular endothelial cells (HRGECs), with no effects on cell viability [116]. Further investigation revealed that Mn-induced HRGEC permeability changes were associated with disruption of adherens junctions, critical structures responsible for maintaining endothelial cell permeability [38]. This was as evidenced by decreased expression of VE-cadherin, a protein crucial for cell junction maintenance, and disrupted localization of VE-cadherin at cell–cell contacts after Mn exposure. The study also investigated the underlying signaling pathways involved in Mn-induced VE-cadherin downregulation, finding that Mn treatment activated the Smad2/3-Snail signaling pathway, leading to decreased VE-cadherin expression in HRGEC [116]. Overall, these findings suggest that Mn exposure disrupts endothelial cell junctions and increases permeability through activation of the Smad2/3-Snail signaling pathway, highlighting a potential mechanism for endothelial dysfunction-induced vascular alterations.

The toxic effects of Mn were also observed in bovine aortic endothelial cells (BAECs) [117]. In contaminated groundwater, Mn is commonly found in conjunction with arsenic, a metal whose long-term exposure is associated with endothelial dysfunction [118, 119]. Because of the frequent simultaneous presence of both metals in contaminated settings, Bunderson et al. [117] tested whether the toxic effects of Mn and arsenic in endothelial cells are additive, where exposure to the mixture would generate an enhanced endothelial cell dysfunction. The authors found that Mn (50 μM) potentiated the toxic effects of arsenic on the viability of BAECs [117]. In addition, the study found that Mn and arsenic independently increased the levels of the reactive biological oxidant peroxynitrite in BAECs [117]. The combination of arsenic and Mn resulted in significantly higher peroxynitrite formation than either metal alone, suggesting an additive effect of these metals on oxidative stress. Moreover, inflammation, a critical factor that impairs the endothelial function, was investigated by measuring the proinflammatory leukotriene E4 (LTE4) [117]. Exposure to arsenic or Mn individually increased LTE4 synthesis in BAECs, with an additive effect observed in the arsenic/Mn combination. Inhibition of NOS by L-NAME resulted in further enhancement of LTE4 synthesis in BAECs exposed to the arsenic/Mn mixture, indicating that this additive effect involves a complex interplay between oxidative stress and NO pathways [117]. Taken together, these findings suggest that Mn enhances the toxicity of arsenic to endothelial cells, exacerbating oxidative stress and inflammatory responses, which may have potential implications for cardiovascular diseases.

On the other hand, protective effects of Mn were observed in studies evaluating endothelial cells exposed to conditions associated with diabetes [120, 121], a metabolic disorder characterized by endothelial dysfunction resulting from inflammation, oxidative stress, and advanced glycation end-products (AGEs) [122]. Because Mn is crucial for the activity of metalloenzymes, such as manganese superoxide dismutase (MnSOD) and is reported to have antioxidant properties at low concentrations [123, 124], Burlet et al. hypothesized that the metal would exert beneficial effects on human umbilical vein endothelial cells (HUVECs) exposed to high glucose to mimic diabetic conditions [121]. The authors found that Mn at 10 μM reduced the secretion of the inflammatory cytokine monocyte chemoattractant protein-1 (MCP-1) in HUVECs exposed to high glucose [121]. In addition, Mn supplementation inhibited high glucose-induced monocyte adhesion to endothelial cells, possibly by downregulating the ICAM-1 expression. This effect was associated with a decrease in reactive oxygen species (ROS) levels, indicating a potential role of Mn at 10 μM in mitigating oxidative stress in endothelial cells. Knockdown of manganese superoxide dismutase (MnSOD) expression did not abolish the inhibitory effects of Mn on monocyte adhesion and ICAM-1 expression [121], suggesting that Mn exerts its protective effects on endothelial function independent of MnSOD activity.

These findings are in line with another study that observed beneficial effects of Mn in BAECs exposed to AGE, compounds formed by non-enzymatic reactions between carbonyl groups of reducing sugars and free amino groups of proteins, lipids, or nucleic acids [120]. Compelling evidence has shown that these compounds contribute to diabetes complications by promoting inflammation and oxidative stress, ultimately resulting in endothelial dysfunction [122]. In this study, reduced BAEC viability was observed in cells exposed to AGE, which was reverted by Mn supplementation at the concentrations of 5, 10, and 20 μM [120]. Mn supplementation (10 μM) also attenuated the increased ROS levels induced by AGE, as well as the AGE-induced expression and nuclear translocation of NF-κB, the primary transcription factor for several proinflammatory cytokines [125]. In addition, supplementation of BAECs with Mn at 10 μM improved the decreased NOS3 expression and activity induced by AGE, even though it did not restore the decreases in NO levels promoted by AGE [120]. Collectively, these findings suggest potential benefits of Mn at low concentrations in mitigating the adverse effects of AGEs on endothelial cells, including the restoration of cell viability, attenuation of oxidative stress, suppression of NF-κB-mediated inflammation, and improvement of NOS3 expression and activity.

**Fig. 1:**
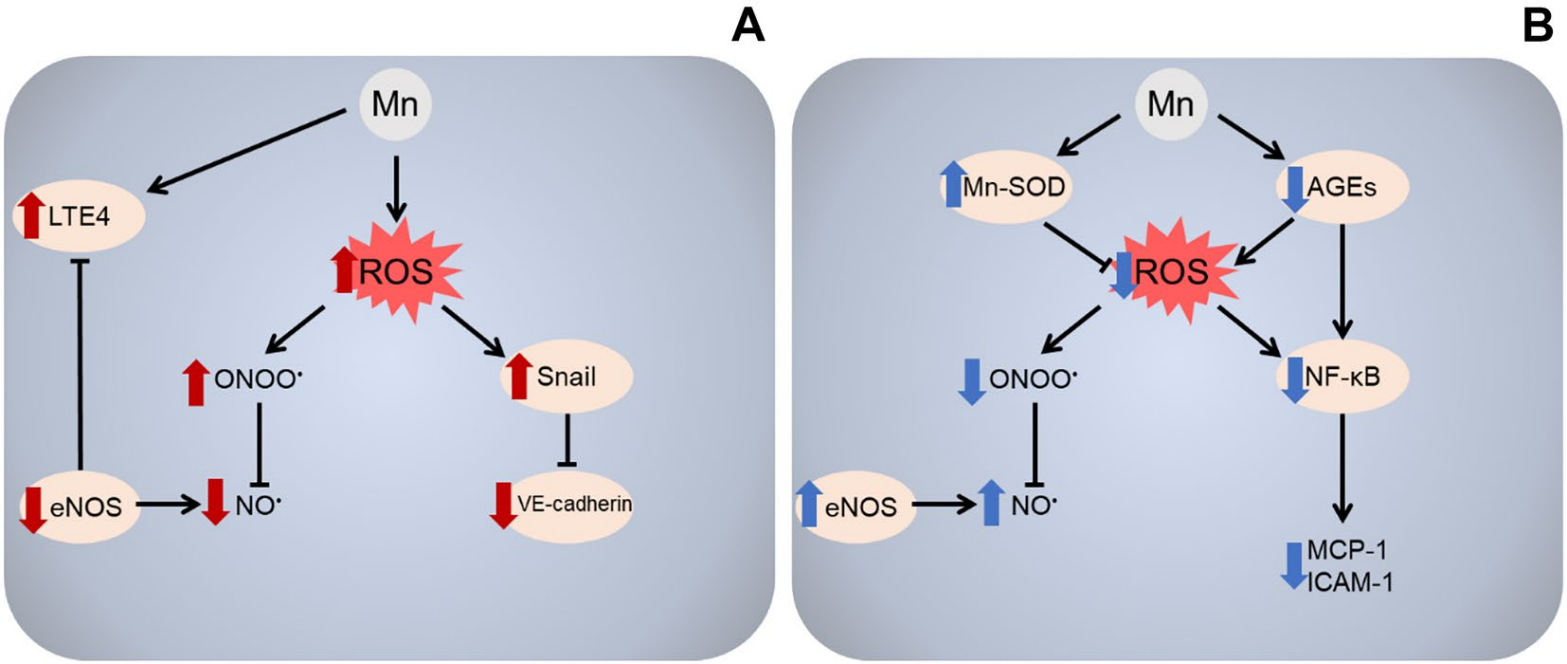
A summary of the molecular mechanisms underlying dual effects of Mn in peripheral endothelial cells. Mn was shown to induce endothelial dysfunction upon overexposure (**a**). Mn-induced ROS overproduction significantly reduces NO bioavailability due to the reaction between NO and superoxide with the formation of highly-reactive peroxynitrite (ONOO) further promoting cell damage. Down-regulation of NOS may be also responsible for a decrease in NO production, ultimately affecting NO-induced vasodilatation. In addition, Mn exposure significantly increases disruption of adherens junctions through down-regulation of VE-cadherin expression via Smad2/3-Snail signaling activation. Finally, Mn exposure promoted proinflammatory response in endothelium through up-regulation of LTE4 biosynthesis, that was at least partially mediated by inhibition of NOS. Due to essential role of Mn as a cofactor of a number of enzymes, Mn was also shown to possess protective effects in endothelial cells (**b**). Mn treatment significantly reduces ROS production through up-regulation of Mn-SOD and inhibition of AGEs formation. Furthermore, Mn also attenuated AGE-induced activation of NF-kB signaling ultimately resulting in reduced production of proinflammatory cytokine MCP-1 and adhesion molecule ICAM-1. Reduction of ROS formation by Mn together with up-regulation of NOS activity significantly increased NO production and bioavailability

Taken together, the findings discussed above suggest a dual impact of Mn on peripheral endothelial cells, indicating that while higher concentrations of Mn exacerbate endothelial dysfunction by inducing oxidative stress, disrupting cell junctions and increasing permeability, lower concentrations may have a protective effect on endothelial function in cells exposed to conditions associated with diabetes, such as high glucose and AGE (Fig. 1).

### Effects of Mn on brain endothelial cells

While Mn plays a crucial role as a cofactor for numerous enzymes, such as glutamine synthetase, superoxide dismutase, and glycosyltransferases [126], a disruption in Mn homeostasis, caused by excessive environmental exposure or impaired Mn trafficking, has been linked to various neuropathologies [6]. Overexposure can result in Mn accumulation in the basal ganglia, the brain region responsible for controlling voluntary muscle movements [127]. This accumulation can lead to conditions such as manganism, hypermanganesemia, and Parkinson’s disease, characterized by impaired motor coordination [6]. Mn can enter the brain from blood plasma by crossing the BBB, which is composed mainly of endothelial cells that have specialized tight junctions [15]. Despite these tight junctions between endothelial cells forming a restrictive barrier, Mn readily crosses the BBB and at high levels may promote neurotoxicity [128]. Consequently, studies have investigated the mechanisms by which Mn is taken up by brain endothelial cells and its impact on their structure and function. Understanding these processes is crucial to elucidate how this metal ultimately accumulates in the brain and contributes to neurological impairment.

Given that the chemical form of metals may influence their absorption, distribution, and toxicity [129], Aschner et al. [130] compared the transport kinetics of various Mn salts (chloride, sulfate, phosphate) in rat brain endothelial (RBE4) cell cultures as a model of the BBB. They found that the initial uptake rate was salt-dependent, with Mn chloride and Mn sulfate having the highest rates, greater than Mn phosphate [130]. Fitsanakis et al. further characterized Mn transport in RBE4 cells, examining temperature, energy, proton, iron and sodium dependence [131]. The study revealed that Mn permeability and flux across the RBE4 cells were significantly dependent on temperature and energy, suggesting an active transport process. Additionally, there was partial dependence on protons, indicating the involvement of proton gradients or proton-coupled transporters. However, Mn transport was independent of sodium, ruling out certain sodium-coupled mechanisms. Consistent with a previous study [132], the authors also found that Mn flux and permeability were lower when RBE4 cells were cultured in astrocyte-conditioned media compared to standard media, implying that astrocyte-derived factors can modulate Mn transport across the BBB [131]. Taking all these findings into account, the authors concluded that the observed properties are consistent with carrier-mediated active transport processes mediating Mn influx across the BBB.

Some metals often share common transport mechanisms facilitated by the same set of carriers [73]. Initial insights into Mn uptake by brain endothelial cells were derived from studies investigating iron transport and absorption, particularly those focusing on the divalent metal transporter-1 (DMT1) and transferrin/transferrin receptor 1 (TfR1) [73]. DMT-1 is expressed at the brain endothelial and functions as a high-affinity transporter for non-transferrin-bound divalent cations, exhibiting a non-selective affinity for various divalent metal ions, including Mn [15, 133]. However, studies in Belgrade rats lacking functional DMT-1 found no difference in brain Mn influx vs controls, arguing against an essential role for DMT-1 [134]. Another carrier expressed at the brain endothelial cells is TfR1, which transport trivalent cations bound to transferrin [73]. While trivalent Mn can bind to transferrin, studies where rat brains were perfused with Mn solutions and experiments measuring Mn cellular uptake have shown that Mn^3+^-transferrin is taken up much more slowly than Mn^2+^ species [135, 136], suggesting that TfR1 might not be a critical Mn carrier mediating Mn influx across the BBB.

Instead, other proteins seem to be more relevant for Mn uptake by brain endothelial cells. In this respect, Steimle et al. investigated the role of ZIP8 (solute carrier family 39 member 8, SLC39A8) and ZIP14 (solute carrier family 39 member 14, SLC39A14), two recently identified members of the Zrt- and Irt-like protein (ZIP) family of metal transporters, on Mn uptake in human brain microvascular endothelial cells (hBMVECs) [137]. They demonstrated that both ZIP8 and ZIP14 facilitate Mn accumulation in these cells in a pH-, lipopolysaccharide-, and bicarbonate-dependent manner, indicative of their activity. Importantly, ZIP8 and ZIP14 were localized and functional at both the apical (blood-facing) and basolateral (brain-facing) membranes of hBMVECs [137], suggesting bidirectional Mn flux across the BBB. In a subsequent study, they evaluated the role of the P-type ATPase secretory pathway Ca2+ATPase1 (SPCA1) in regulating Mn uptake in hBMVECs [138]. SPCA1, encoded by the *ATP2C1* gene, is a protein localized in the Golgi apparatus that functions as a calcium pump, transporting calcium from the cytosol into the lumen of the Golgi [139]. While SPCA1 has also been implicated in Mn import into the Golgi [140], its importance in Mn uptake in brain endothelial cells remained unknown. In their study, Steimle et al. found that SPCA1 modulates cytoplasmic calcium levels in hBMVECs, which in turn regulates the plasma membrane localization and activity of the Mn transporters ZIP8 and ZIP14 [138]. Specifically, knockdown of SPCA1 increased cytoplasmic calcium and enhanced Mn uptake by promoting the membrane trafficking of ZIP8 and ZIP14. Conversely, overexpression of SPCA1 or a gain-of-function mutant decreased cytoplasmic calcium and reduced Mn accumulation [138]. These findings suggest that SPCA1 indirectly regulates Mn homeostasis in hBMVECs by modulating cytoplasmic calcium levels, which control the membrane localization and activity of ZIP8 and ZIP14, the primary Mn transporters identified in their previous study.

More recently, Wu et al. investigated the role of the cytochrome P450 enzyme CYP1B1 in Mn-induced toxicity using in vitro experiments with human cerebral microvascular endothelial cells (hCMEC/D3), an in vitro model of the BBB [141]. The cytochrome P450 enzyme CYP1B1, an extrahepatic isoform involved in arachidonic acid metabolism in the brain cortex and cerebellum, is abundantly expressed in brain microvascular endothelial cells, comprising 77% of CYP isoforms in the brain microvasculature [142–145]. However, its role in Mn neurotoxicity was previously unknown. In their study, Wu et al. found that Mn exposure decreased CYP1B1 mRNA levels in hCMEC/D3 [141]. Overexpression of CYP1B1 in these cells increased the mRNA levels of tight junction proteins (zonular occludens-1, occludin, claudin-1) and PPARγ, a nuclear hormone receptor and a ligand-activated transcription factor that regulates tight junctions. Indeed, overexpressing PPARγ in hCMEC/D3 upregulated tight junction protein mRNAs [141], suggesting that PPARγ may be involved in how CYP1B1 regulates tight junction expression. Therefore, they investigated the potential role of the PPARγ in mediating the effects of CYP1B1 metabolites on tight junctions under Mn exposure conditions. They observed that CYP1B1-derived metabolites such as 5-Hydroxyeicosatetraenoic acid (5-HETE) and 15-HETE from arachidonic acid increased PPARγ transcriptional activity and prevented the Mn-induced downregulation of PPARγ and tight junction proteins in hCMEC/D3 cells [141]. This indicates that CYP1B1-derived HETEs protect against Mn disruption of the BBB by activating PPARγ signaling and preserving the expression of tight junction components. The results indicate a protective role of brain endothelial CYP1B1 against Mn-induced BBB disruption and suggest it as a potential therapeutic target.

**Fig. 2:**
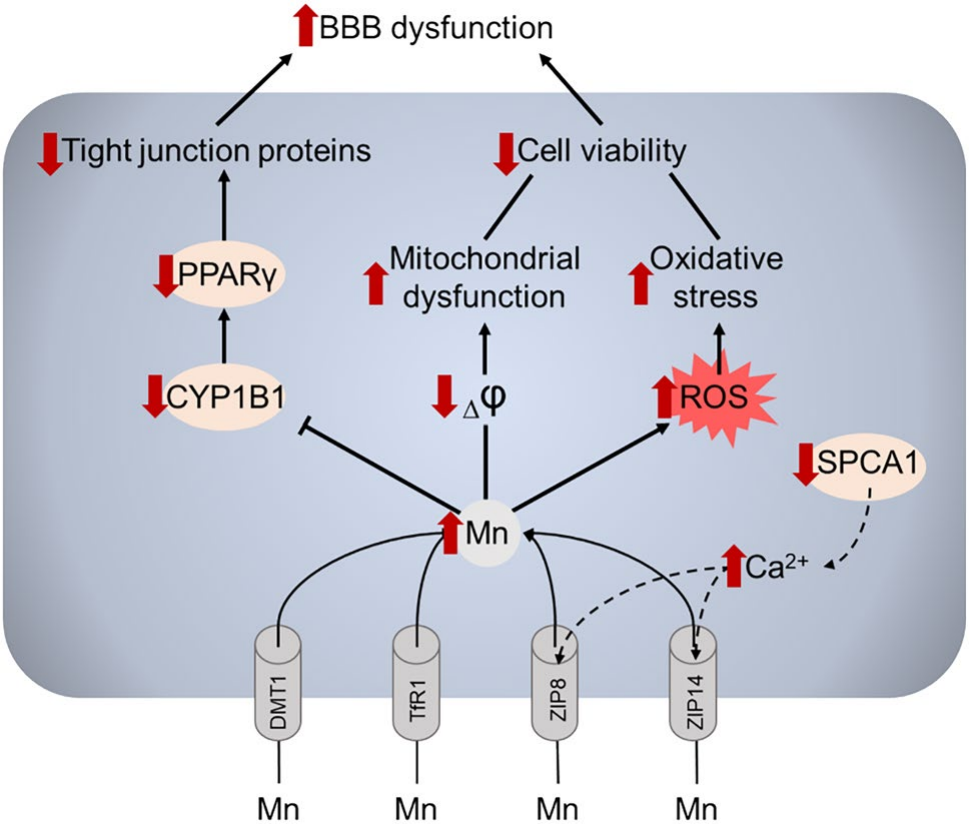
A summary of the effects of Mn in blood–brain barrier endothelial cells

In their studies on rat brain endothelial (RBE4) cells as an in vitro model of BBB, Marreilha dos Santos et al. demonstrated the cytotoxic effects of Mn exposure [146, 147]. Exposure to MnCl_2_ or MnSO_4_ (200 or 800 μM) for 4 or 24 h led to a significant, concentration-dependent decrease in RBE4 cell viability, which was further exacerbated by pre-treatment with glutathione (GSH) depletors such as diethylmaleate (DEM) or buthionine sulfoximine (BSO), suggesting oxidative stress involvement [147]. Mn exposure also increased levels of F2-isoprostanes, markers of lipid peroxidation, and significantly decreased the mitochondrial membrane potential, with DEM pre-treatment exacerbating this effect [147]. These findings indicate that Mn induces mitochondrial injury in brain endothelial cells, leading to impaired energy metabolism and redox status. In another study, they found that 24-h exposure to MnCl_2_ or MnSO_4_ (50–800 μM) significantly decreased RBE4 cell viability in a concentration-dependent manner [146]. Mn exposure also reduced intracellular GSH levels, but co-exposure with antioxidants such as N-acetylcysteine (NAC) or Trolox effectively attenuated the Mn-induced decrease in cell viability, confirming oxidative stress mechanisms [146]. Taken together, these studies suggest that brain endothelial cells are direct targets of Mn neurotoxicity, where oxidative stress and mitochondrial damage may compromise BBB integrity, facilitating the entry of Mn into the brain and contributing to neurotoxicity (Fig. 2).

Mn is transported to BBB endothelial cells via DMT1, TfR1, ZIP8, and ZIP14, although the existing data on the contribution of the particular transporters to cellular Mn uptake are scarce. It has been also demonstrated that SPCA1 localized in the Golgi apparatus modulates membrane trafficking of ZIP8 and ZIP14 through regulation of intracellular Ca2 + levels. Upon overaccumulation, Mn promotes ROS overproduction together with mitochondrial dysfunction, altogether resulting in reduction of endothelial cell viability. In addition, a number of toxic effects of Mn in the endothelial cells including reduction of tight junction proteins. Together with reduction of cell viability, alterations in tight junctions may promote BBB dysfunction induced by Mn overexposure.

## Endothelial barriers and Mn toxicity: in vivo studies

In addition to the in vitro findings discussed above, some studies have used in vivo approaches to elucidate the role played by endothelial cells and barrier structures in mediating Mn toxicity in organs systems, such as central nervous system and kidneys. For instance, Gao et al. investigated the impact of Mn overexposure on the glomerular filtration barrier in the kidneys, focusing on the role of the glomerular endothelium and the adherens junction protein VE-cadherin in mediating Mn-induced glomerular permeability changes [116]. In their study, Sprague Dawley rats were intraperitoneally injected with Mn chloride (25 mg/kg body weight) every 48 h for 3 months to mimic Mn overexposure. Although no significant morphological changes or inflammatory cell infiltration were observed in the glomeruli of Mn-treated rats compared to controls, there was an increase in protein exudate from the glomeruli, suggesting impairment of the glomerular filtration barrier. This was accompanied by a significant decrease in both mRNA and protein levels of VE-cadherin, a key component of endothelial adherens junctions, in the glomeruli of Mn-exposed rats. Immunohistochemistry further confirmed the downregulation of VE-cadherin specifically in glomerular cells following Mn treatment [116]. These findings highlight the importance of the glomerular endothelium in mediating the effects of Mn toxicity on the kidney, as the loss of endothelial VE-cadherin and consequent disruption of cell–cell junctions likely contributed to the increased glomerular permeability and proteinuria observed in vivo.

Other studies, such as the one performed by Liu et al., have focused on the impact of Mn exposure on BBB disruption in vivo [148]. They found that mice exposed to Mn have perivascular edema, hypertrophic endothelial cells, and diffusion of serum albumin into the perivascular space in their striatum and globus pallidus, indicating compromised BBB integrity. Fluorojade staining revealed dying endothelial cells within edematous vessels, suggesting that the cerebral microvasculature is a primary target of Mn toxicity. Furthermore, many apoptotic striatal interneurons expressing neuronal NO synthase, choline acetyltransferase, and enkephalin were located proximally to capillaries, suggesting that BBB disruption and endothelial cell injury may predispose nearby neurons to degeneration [148]. The authors propose a mechanism of gliovascular dysfunction, where endothelial cell damage triggers astrocyte activation, inducible NO synthase expression, and subsequent NO-mediated injury to striatal interneurons. These findings highlight the critical role of the BBB and cerebral microvasculature in mediating Mn-induced neurotoxicity and neuronal degeneration within the basal ganglia.

Considering the deleterious effects of Mn on BBB, studies have investigated the in vivo mechanisms underlying the Mn uptake by brain and their relationship with the BBB. In this regard, the in vitro findings showing the role of ZIP8 (SLC39A8) in Mn uptake by brain endothelial cells discussed in the previous section [137] were validated in an in vivo study performed by Liu et al. that investigated the role of the ZIP8 in brain Mn uptake and accumulation using Slc39a8 inducible knockout (iKO) mice [149]. Radiotracer studies using ^54^Mn revealed that Slc39a8 iKO mice had markedly diminished ^54^Mn uptake in the brain compared to controls, indicating that SLC39A8 is essential for brain Mn uptake [149]. Importantly, the decreased ^54^Mn uptake in the brains of Slc39a8 iKO mice was associated with efficient inactivation of Slc39a8 in isolated brain microvessels, but not in choroid plexus [149]. This suggests that ZIP8 (SLC39A8) mediates brain Mn uptake primarily via the BBB endothelium rather than the choroid plexus. The findings highlight the crucial role of this transporter in the brain microvasculature endothelial cells in regulating brain Mn homeostasis in vivo.

In addition to investigating the involvement of CYP1B1 in Mn-induced toxicity in brain endothelial cells [141], as discussed in the previous section, the study by Wu et al. also investigated the role this protein in Mn-induced neurotoxicity in vivo using wild-type and CYP1B1 knockout mice [141]. Mn exposure increased brain Mn levels, exacerbated motor deficits, and promoted α-synuclein accumulation in dopaminergic neurons of the substantia nigra in wild-type mice [141]. These neurotoxic effects were aggravated in CYP1B1 knockout mice, which exhibited higher brain Mn accumulation. In vivo multiphoton microscopy revealed reduced microvascular length, extravasation of fluorescent dextran, and co-localization of endothelial marker CD31 with fibrin in the cortex of CYP1B1 knockout mice, indicating BBB disruption. Immunohistochemistry showed decreased levels of the tight junction protein zonular occludens-1 in cortical and striatal microvessels of these mice [141]. These findings highlight the importance of brain endothelial CYP1B1 in maintaining BBB integrity, as its deficiency exacerbated Mn-induced BBB disruption, increased brain Mn accumulation, and worsened dopaminergic neurodegeneration, underscoring the critical role of the endothelium in mediating Mn neurotoxicity.

Taken together, the studies summarized above consistently highlight the in vivo relevance of the endothelial cells forming the BBB and the glomerular filtration barrier in mediating the toxic effects of Mn overexposure in the brain and kidneys, respectively. The studies underscore the vulnerability of the endothelial barriers in these organs to Mn toxicity, suggesting that preserving endothelial integrity may prevent Mn-induced organ damage.

In addition to the studies in animal models, some epidemiological studies have reported associations between Mn exposure and disorders linked to endothelial dysfunction, such as high blood pressure. A Brazilian study observed that higher diastolic blood pressure was associated with elevated blood Mn levels in a group of women [150]. Another study examined 386 women to investigate the effects of prenatal manganese exposure on gestational blood pressure and observed that early pregnancy blood Mn levels were correlated with blood pressure throughout gestation, showing a significant association between the odds of pre-hypertension and blood Mn levels [151]. In a study of adults aged over 65 years, an association was reported between blood manganese concentrations and 24-h based brachial and central blood pressure [152]. These findings are further supported by larger-scale studies. A prospective cohort study involving 12,177 participants from China observed a significantly higher risk of new-onset hypertension in participants with manganese intake ≥ 6.0 mg/day compared to those with intake < 6.0 mg/day [153]. Taken together, these findings highlight the potential impact of Mn exposure on increased blood pressure regulation across different populations, which might be mediated, at least in part, through endothelial cell dysfunction, which plays a crucial role in vascular health and blood pressure control.

## Concluding remarks

The vascular endothelium, a crucial component of the cardiovascular system, plays a pivotal role in maintaining homeostasis by regulating various physiological processes, including vascular tone, blood coagulation, and immune response. Endothelial cells are highly sensitive to environmental factors, and their dysfunction has been implicated in the pathogenesis of numerous diseases. Mn has been shown to exert both beneficial and detrimental effects on endothelial function, depending on its concentration and the specific organ system involved.

In vitro studies have demonstrated that Mn exposure can lead to endothelial cell cytotoxicity, increased permeability, and disruption of cell–cell junctions in peripheral endothelial cells. These effects are mediated through various mechanisms, including oxidative stress, mitochondrial damage, and activation of signaling pathways such as Smad2/3-Snail. Conversely, low concentrations of Mn have been shown to protect endothelial cells from the deleterious effects of high glucose and AGE, suggesting a potential therapeutic role for Mn in diabetes-associated endothelial dysfunction.

In the central nervous system, Mn can cross the BBB and accumulate in the brain, leading to neurotoxicity. Several transport mechanisms have been identified for Mn uptake by brain endothelial cells, including the solute carriers ZIP8 and ZIP14, as well as SPCA1. Mn exposure has been shown to impair BBB integrity by disrupting tight junctions and increasing permeability, which may facilitate the entry of neurotoxic substances into the brain. Interestingly, the cytochrome P450 enzyme CYP1B1, expressed in brain endothelial cells, has been found to protect against Mn-induced BBB disruption by activating PPARγ signaling and preserving tight junction expression.

In vivo studies have corroborated the findings from in vitro models, highlighting the importance of endothelial barriers in mediating Mn toxicity. Mn exposure has been shown to disrupt the glomerular filtration barrier in the kidneys by downregulating the adherens junction protein VE-cadherin, leading to increased glomerular permeability and proteinuria. In the brain, Mn overexposure has been associated with BBB disruption, endothelial cell injury, and neuronal degeneration in the basal ganglia, emphasizing the critical role of the cerebral microvasculature in Mn neurotoxicity.

In conclusion, the endothelium serves as a critical target for Mn toxicity, with both peripheral and brain endothelial cells exhibiting vulnerability to Mn-induced dysfunction. The delicate balance between the essential physiological roles of Mn and its potential toxicity underscores the importance of maintaining optimal Mn homeostasis. Further research is needed to elucidate the precise mechanisms underlying Mn-induced endothelial dysfunction and to develop targeted therapeutic strategies to prevent or mitigate the adverse effects of Mn overexposure on the cardiovascular and central nervous systems. Exploring the potential protective effects of low-dose Mn supplementation on endothelial function in specific pathological conditions, such as diabetes, may also provide novel insights into the therapeutic applications of this essential trace element.
